# A Feasibility Study of an Extrusion-Based Fabrication Process for Personalized Drugs

**DOI:** 10.3390/jpm10010016

**Published:** 2020-03-04

**Authors:** Ilhan Yu, Roland K. Chen

**Affiliations:** School of Mechanical and Materials Engineering, Washington State University, Pullman, WA 99164, USA; ilhan.yu@wsu.edu

**Keywords:** personalized medicine, manufacturing, drug delivery, controlled release, 3D printing

## Abstract

Developing a high-efficiency manufacturing system for personalized medicine plays an important role in increasing the feasibility of personalized medication. The purpose of this study is to investigate the feasibility of a new extrusion-based fabrication process for personalized drugs with a faster production rate. This process uses two syringe pumps with a coaxial needle as an extruder, which extrudes two materials with varying ratios into a capsule. The mixture of hydrogel, polyethylene glycol (PEG), hydroxypropyl methylcellulose, poly acrylic acid and the simulated active pharmaceutical ingredient, Aspirin, was used. To validate the method, samples with different ratios of immediate release (IR) and sustained release (SR) mixtures were fabricated. The results of a dissolution test show that it is feasible to control the release profile by changing the IR and SR ratio using this fabrication setup. The fabrication time for each capsule is about 20 seconds, which is significantly faster than the current 3D printing methods. In conclusion, the proposed fabrication method shows a clear potential to step toward the feasibility of personalized medication.

## 1. Introduction

Personalized medicine, a new transformative way for medication, calls for the treatment of a patient by delivering the right drugs with the right dose to the right location at the right time [1]. Providing personalized dosing, ensuring the right dose is one major component for personalized medicine [2,3,4,5]. Under the same medication dose regimen, distinct drug reactions can occur, given high variations in each individual’s metabolizing capacity, genetic profile and age [6,7,8,9]. It is reported that inappropriate dosing causes about 75% to 80% of adverse drug reaction cases [10]. The issue of inappropriate dosing is magnified with narrow therapeutic index (NTI) drugs, as small changes in concentration in the system could cause a difference between effective doses and an adverse toxic effect [11]. NTI drugs require highly individualized dosing [7,12], monitoring and the tailoring of dosages for better and safer medication [13]. Personalized or individualized dosing offers several advantages, such as the ability to control different drug release kinetics, and the personalized dosing of narrow therapeutic windows medications, that guides a safer and more effective therapeutic solution [14]. Despite these advantages, there are still a number of challenges that need to be overcome, in order to achieve personalized medicine. One most notable challenge is shifting the current manufacturing system in the pharmaceutical field to one that can offer a customized product.

Unlike the current “one-size-fits-all” drugs, personalized drugs and medications need different manufacturing systems than the current mass production system. First, the manufacturing method needs to offer a high level of flexibility in regards to controlling the dosage as well as the selection of materials [2]. Second, the end processes of fabrication requires “on-demand and on-site” capability [14]. 

Currently, the manufacturing of drugs relies upon mass production systems, and are represented by tablets and capsules that are designed for highly centralized, large batch-based production [15,16]. This mass production system is efficient to produce a “one-size-fits-all” drug, but the system is not flexible enough for customizing [17]. To overcome the customization issue, simple solutions, such as crushing the tablet into halves or quarters, or opening the capsule, has been offered as options to control the dose for each individual [18,19]. However, this customization process not only fails to offer accurate and precise controls of the dose, but often requires a pharmaceutical technician to handle these tasks, which takes time and money [20]. Research that modifies the existing mass production system of compressing and coating into multilayered tablets has been done, allowing for the specific tailoring of the dosage [21,22,23]. However, this mass production-based system modification still uses similar equipment and steps designed for the mass production system. The modification often takes an even longer time than crushing the tablet, and due to the size and expense of equipment, the feasibility to achieve “on demand and on-site” is very low. Previous studies [21,22,23] indicate that re-designing, or modifying, the current mass production system is not suitable to achieve higher flexibility “on-demand and on-site”. Thus, personalized medication demands a paradigm shift, rather than a modification of the current production and manufacturing system.

Three dimensional (3D) printing shows the most potential as the technology that will shift the paradigm of the fabrication of personalized drugs [14,24]. Researchers have used various types of 3D printing methods to fabricate the dosage form. The most common method is fused filament fabrication (FFF) [17,25,26,27,28]. Goyanes et al. proposed a new direct powder extrusion technology, which eliminated the need to use materials in the filament form, in order to fabricate drug products. However, the throughput of this process is similar to the FFF process at 2 to 3 minutes per printlet [29]. Other methods, such as an inkjet [20,30], extrusion [31] and powder type printing [32], have been studied as well. Fabricating the dosage form of a personalized drug with the 3D printing method offers a clear advantage on tailoring multiple drugs, controlling the dose manufacturing complex geometry structure, and its modification. 3D printing has yet to make an impact in clinical practice, due to the fact that the fabrication of customized drugs takes too long, regardless of the printing method. For example, current research shows printing 28 tablets in around 8 mins [33], but restricted by one material. The common 3D printing methods produce only 10–50 tablets per hour [15]. Both the long printing time and slow production rate compromise the “on demand” capability [25]. Furthermore, a more cost-effective fabrication system can be more widely acceptable for better “on-site” capability [6].

Although 3D printing technology has shown great potential so far regarding customization, a faster fabrication process, but yet more affordable equipment, needs to be developed. The goal of this study is to develop a process to increase production rates, while being able to control the dose and combination of these drugs. Thus, we propose a new fabrication system based upon coaxial needle extrusion directly into a capsule, to create two or more combinations of controlled dosing of drugs. In this study, we first characterized material behaviors to determine processing parameters, developed the fabrication system, and lastly, performed in-vitro drug dissolution tests of capsules with different combinations of immediate release (IR) and sustained release (SR), in order to validate the feasibility of the proposed system and method.

## 2. Method

### 2.1. Material

The material was selected based on the demonstration of the drug fabrication and verification of equipment design. Major material used in this research was slightly modified from previous research [31,34]. This combination of material and drug is not designed for any actual therapeutic purpose. The actual active drug or binder varies by application. Hydroxypropyl methylcellulose (HPMC), HPMC2910 (HPMC E4M) and HPMC2208 (HPMC K100M) and Aspirin, (O-Acetylsalicylic acid, ASA) were purchased from VWR (Radnor, PA, USA).

HPMC 2910 contains more methoxy groups, and usually has a lower viscosity as an aqueous solution than does HPMC 2208. HPMC2910 was used for immediate release, and HPMC2208 was used for sustained release. For controlled release by different pH levels, Poly acrylic acid (Carbopol® 974P NF, PAA) was used [31,35], and it was gifted from Lubrizol LifeSciences (Cleveland, OH, USA). To control the pH for the drug release test, hydrochloric acid (HCL) 37%, purchased from EMD Milipore (Burlington, MA, USA) and sodium phosphate tribasic (TSP), were used. Polyethylene glycol (PEG, #202452), purchased from Sigma Aldrich (St. Louis, MO, USA) is widely used as oral drug delivery [36,37,38]. In this research, PEG was used to increase the hydrophilicity as previous research indicated [34]. The HPMC capsule (#00 size) was purchased from PurecapsUSA (Philmont, NY, USA). In this study, ASA was used for the active drug. ASA was ground by mortar before mixing. HPMC and PAA were used for the hydrophilic matrix, and PEG was used as a binder. 

#### 2.1.1. HPMC Gel Preparation

Two different HPMC gels were prepared according to the previous study [39]. 90 °C of bio-grade ultrapure water (18 MΩ) was added to HPMC2910, and stirred until no powder or particle was observed. Cold temperature (5 °C) ultrapure water was added until 1% (*w*/*v*) total concentration of HPMC. The mixture started forming as a gel as the temperature dropped. The gel was stirred again for homogeneity. HPMC2208 was also prepared using the same method. Both HPMC gels were kept at 5 °C for 24 h until they were used for the further experiment.

#### 2.1.2. IR and SR Mixture Preparation

The compositions of different IR and SR mixtures are listed in Table 1 and Table 2, respectively. For IR, ASA was mixed with PEG and HPMC. For SR, ASA powder was mixed with PEG and PAA powders. The mixture was shaken and mixed together for homogenous distribution, and then HPMC gel, prepared in the previous step, was added by weight ratio. This mixture of gel and powders was stirred and mixed until a homogeneous paste form was achieved. This paste was loaded into 5 mL syringes, then kept at 60 °C in the incubator. Bubbles that formed inside of the syringe were removed before use.

### 2.2. Characterization of Material Extrusion Behavior

To determine the optimal extrusion parameters, it is critical to understand how the materials behave during the extrusion process. First, a rheology test was performed to study the viscosity of the materials at different temperatures. Then, actual extrusion through the needle was performed to determine the optimal condition of extrusion. The detail about each process is further explained below.

#### 2.2.1. Viscosity Test

The viscometer (Discovery HR2, TA instrument, New Castle, DE) with a 25 mm testing plate was used. The test was performed using 1% strain and a strain rate of 10 rad/s. A 1000 μm gap of parallel plate was used. Tests started at a temperature of 60 °C, and data was collected from 60 °C to 35 °C with a 1 °C step. The range of temperature was selected from around the body temperature to the melting point of PEG [40]. A total of six samples were tested. IR-A, -B, -C and SR-A, -B, -C. Tests were stopped when the mixture became completely solid, and then we stopped the machine. 

#### 2.2.2. Extrusion Temperature Test

The viscosity test provides a general idea of the material behavior. However, in the actual experimental or fabrication setup, air convection and the ambient environment may cool down the material in the system. This cooling effect can cause clogging in the needle, and results in an uneven extrusion rate. Therefore, a better understanding about the material behavior in the needle extrusion system is still needed. To observe how the cooling effect during the extrusion process affects the result, the extrusion temperature test was performed. Mixtures with three different concentrations, kept in the syringe, were preserved in the incubator with the target temperature. The testing condition ranged from 60 °C to 40 °C with a 5 °C temperature step. The temperature range was selected based on the viscosity test. Different needle sizes (gauges 8, 10, 12 and 14) were used. The syringe was placed in the syringe pump (FUSION200, CHEMXY, Stafford, TX, USA), and material was extruded with a 6 ml/min extrusion speed. The results were categorized into success, extrusion with chunks or clogging, and fail. Success is considered if the extrusion was smooth, and had a constant extrusion rate and a linear extrudate shape. Extrusion with chunks or clogging is for the cases in which the resulting extrusion rate is not constant, or the extrudate shape is not constant. A fail is represented by the syringe pump stalling, causing the process to stop. 

### 2.3. Fabrication Setup and Experimental Procedures

#### 2.3.1. Fabrication Setup

**Figure 1:**
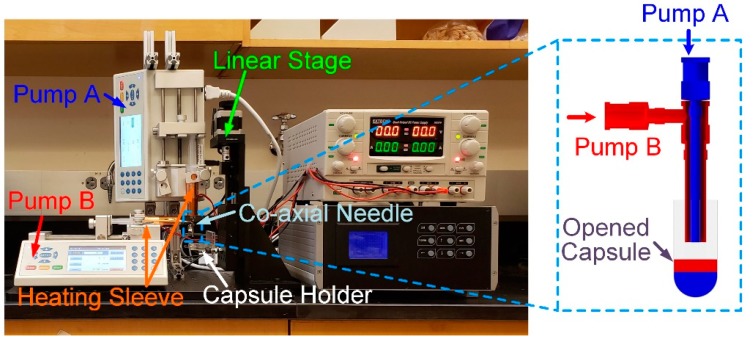
Overview of the fabrication setup. Capsule holder (white box), linear stage (green box), coaxial needle (yellow box), heating sleeve (black arrow).

Figure 1 shows the overview of the fabrication setup. Two syringe pumps were installed vertically (Pump A) and horizontally (Pump B). The inner needle and outer needle were connected to Pump A and Pump B, respectively. Each syringe was covered by a heating sleeve to maintain the temperature at a desired level. Both materials were extruded into a size #00 capsule, held by a capsule holder. The capsule was opened before it was placed into a capsule holder. The capsule holder was attached to a linear stage (XYZ motorized stage, Optics focus, Beijing China). During the extrusion process, the linear stage moved the capsule holder together with the capsule down simultaneously, so that the tip of the coaxial needle always located right above the surface of the extruded material. A LabVIEW program (National Instrument, Austin, TX, USA) was used to control the linear stage and syringe pump. This is an open-loop control system, in which all parameters including the total volume of extrusion, moving distance and speed, were pre-calculated.

#### 2.3.2. In-Capsule Extrusion

**Figure 2:**
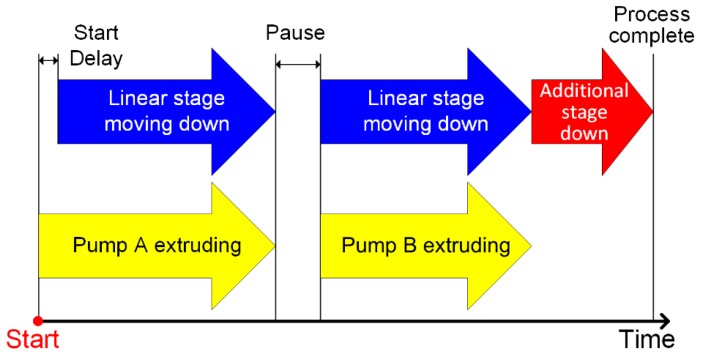
General extrusion process to the capsule: The extrusion time and the linear stage control period depend on the ratio of the two materials. An additional stage (red arrow) provides enough distance between needle and the capsule for replacement of the capsule.

Figure 2 illustrates the sequence of operation in the fabrication system. The process starts with extrusion by Pump A, and with a short delay, the linear stage moves as the capsule is filled, so that the tip of the needle stays above the surface of the extruded material. Once the Pump A extrusion is done, a short 1-second pause is implemented to allow finishing material extrusion due to residual pressure in the syringe before Pump B starts, which follows the same sequence as that of Pump A. The extrusion rate was 6 mL/min for both needles. The period of extrusion for each pump depends on the mixture combination, as listed in Table 3. For each capsule, a total of 0.9 ml of material was extruded into the capsule. The total travel distance of the linear stage is 2 cm, with a speed of around 2 mm/sec. Once the extrusion process is completed, the linear stage moves down an additional 2 cm to allow more clearance for removing the capsule from the holder. The cap of the capsule was re-capped. The total amount of time to fabricate one capsule was 15 seconds. 

#### 2.3.3. In Vitro Dissolution Test 

An in-vitro drug dissolution test was performed to examine the difference in the drug release profiles of capsules with different combinations of IR and SR materials. This test was performed to confirm the possibility of controlled release, so only one set, IR-C and SR-C of the sample, was tested. Each capsule was prepared using a combination of IR-C and SR-C for easier extrusion. Table 3 shows a total of five combinations used for the test. In the 0.9 mL volume of the capsule, the ratio of SR and IR varies from 0% to 100 %. Each of the five combinations was repeated three times. The dissolution test followed the protocol modified from a previous research [41]. The test was designed to mimic two phases. In the first phase (i.e., the acid phase), the tablet was submerged in 750 mL of 0.1M HCL solution at 37 °C, with sink conditions maintained throughout the test. To prevent these tablets from sinking or floating, the tablet was kept in a container made of aluminum mesh. The solution was circulated by a magnetic stirrer at 60 RPM. 1 mL of solution was collected every 15 minutes during the first two hours. At the two-hour mark, the second phase (i.e., the buffer phase) was started. An additional 0.2 M of 250 mL of trisodium phosphate dodecahydrate (TSP) was added to increase the pH to 6.8 pH. The pH was checked with a pH meter. 1 ml of sample was collected every hour until the end of the 8-hour testing period. The collected samples were kept in cuvettes until they were analyzed with a UV spectrophotometer (Evolution™ 260 Bio UV-Visible Spectrophotometer, Thermo Scientific, Waltham, MA, USA), so as to measure the accumulated ASA release. The peak λ_max_ at 280 nm and 265 nm were used for the acid phase and the buffer phase, respectively. 

1M of blank HCL and 0.2M TSP.12H2O added HCL was used for calibration, and the accumulated drug release rate was calculated by the UV absorbance level. 

#### 2.3.4. Scanning Electron Microscope (SEM)

A Scanning Electron Microscope (FEI SEM Quanta 200 F, Hillsboro, OR, USA) was employed to observe the microlevel structure of the sample. The capsule form of the sample was cut in half, and the cross-sectional area was observed. The analysis was done without any coating on the surface. The low vacuum setting with a beam strength of 20.00 kV was used. The main interest of this study was to observe porosity and surface roughness inside of the structure, and compare the difference in the SR region and the IR region.

## 3. Results

### 3.1. Viscosity and Extrusion

**Figure 3:**
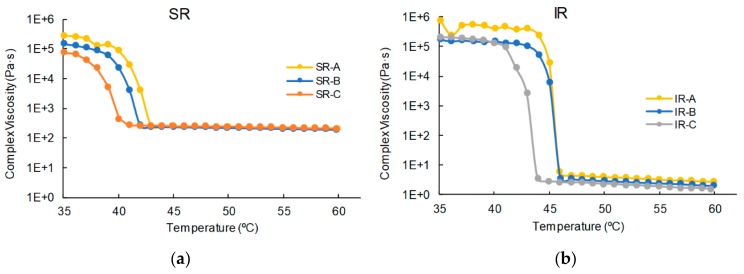
Viscosity test results: (**a**) SR-A, -B and -C and (**b**) IR-A, -B and -C.

**Figure 4:**
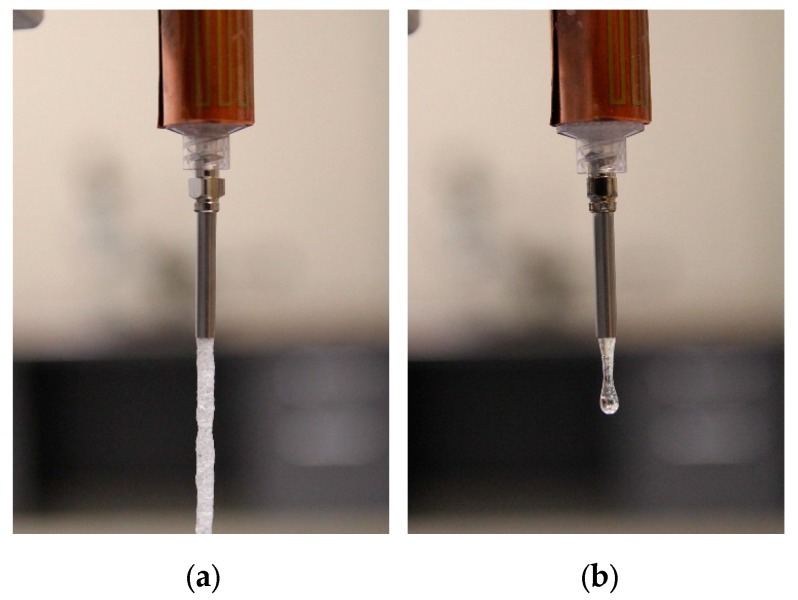
Difference between SR mixture (**a**) and IR mixture (**b**) in terms of extrusion.

Figure 3 shows the result of the viscosity tests. In both SR and IR, the graph indicates that a glass transition temperature (T_g_) region exists. The SR mixture did not show a significant difference between SR-A, -B and -C. Above 44 °C, SR-A, -B and -C show a complex viscosity of around 200 Pa·s. It suddenly increases at around 40 °C in all three mixtures. For the IR mixture, a complex viscosity at higher temperatures is observed, and the viscosity value (less than 10 Pa·s) is lower than that of SR in all three mixtures, IR-A, -B and -C. The glass transition region was observed to be 44 to 46 °C for IR. The difference of complex viscosity at higher temperatures is demonstrated in the extrusion test as well. Figure 4 shows the difference between the SR mixture and IR mixture under the same extrusion condition. The extruded SR mixture forms a cylindrical shape of the uniform structure (Figure 4a). On the other hand, the IR mixture shows that the mixture instantly becomes a drop-by-drop consistency as soon as it leaves the needle (Figure 4b).

**Figure 5:**
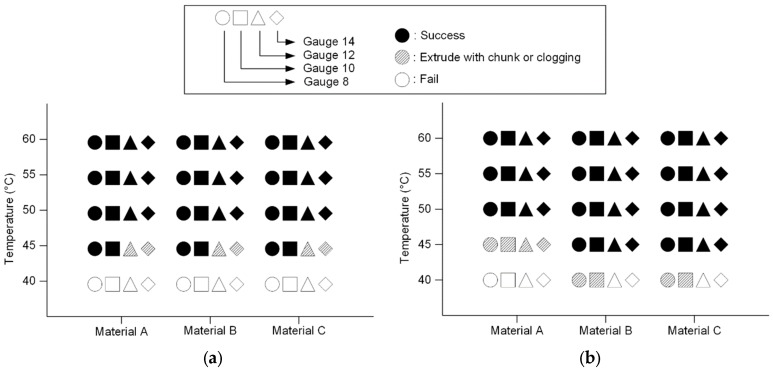
Manufacturability of different material mixtures at various temperatures: (**a**) SR and (**b**) IR. At 40 °C, both IR and SR mixture failed. Failure or clogging was observed until 45 °C in both mixtures, but from 50 °C, the extrusion was uniform and smooth.

Figure 5 shows the results of the extrusion test. Figure 4 and Figure 5 indicate SR has a much higher viscosity at the same temperature. A higher viscosity indicates that SR extrusion is not as smooth as IR. However, if the material solidifies faster than its extrusion speed, it can cause clogging or failure. The trend of failure shows the difference between SR and IR. SR failed in smaller needles, due to a higher viscosity at relatively lower temperature (45 °C), but IR failed mostly due to the decrease in temperature. For example, all cases of Material A failed, regardless of the needle size at 45 °C. It can be concluded that it is safe to extrude at above 50 °C, regardless of the mixture type. At 50 °C or above, the needle size was not a factor for clogging. Thus, selecting the needle combination for the coaxial needle was purely determined based on the sizes of the needles. In this study, the gauge 8 needle was used for the outer needle. The largest size of inner needle that fits the gauge 8 outer needle was the gauge 12, and thus the combination of gauge 8 and gauge 12 was used in all subsequent tests.

### 3.2. Fabricated Capsules

**Figure 6:**
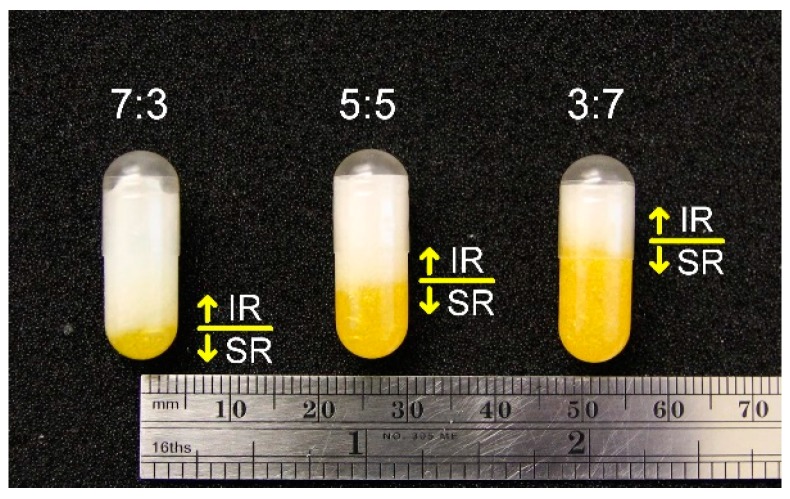
Fabricated capsules with different IR (white) and SR (yellow) mixtures. From left to right: I7S3, I5S5 and I3S7.

**Figure 7:**
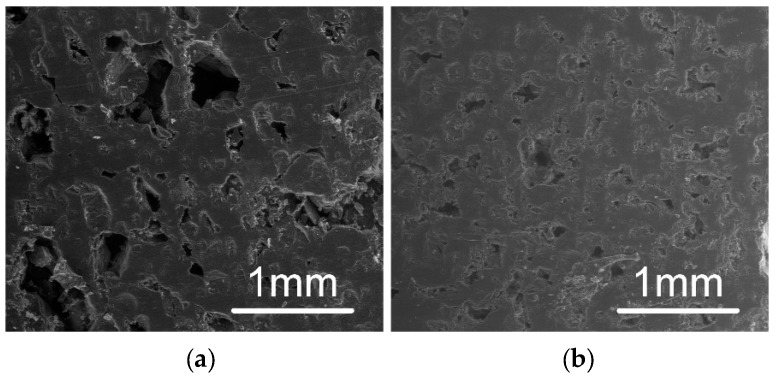
Scanning electron microscopy (SEM) images of the surfaces of (**a**) SR layer and (**b**) IR layer. The SR layer shows more porosity with rough texture. The IR layer shows much less porosity and fine texture.

Figure 6 shows examples of fabricated capsules with the different ratios of SR and IR. The SR (the yellow portion) was extruded first, and then the IR (the white portion) filled up the rest, with a total volume of 0.9 mL. “One concern about this process was if the material extruded at an elevated temperature would damage or dissolve the capsule. As shown in Figure 6, no significant changes and dissolving were observed after the extrusion process was complete.” All capsules have the same total volume, as indicated by the consistent top surface level of the IR layer. Each capsule was fabricated in about 15 seconds. From the SEM images as shown in Figure 7, the texture of the SR layer is rougher than that of the IR layer. Pores at SR appear bigger as Figure 7 indicates. This difference was observed through all the samples between the SR group (-A, -B and -C) and the IR group (-A, -B and -C). However, between SR-A, -B, -C or IR-A, -B, -C, there was not much difference. As Figure 4 indicated, SR contained more air bubbles, causing more porosity.

### 3.3. Dissolution Test

**Figure 8:**
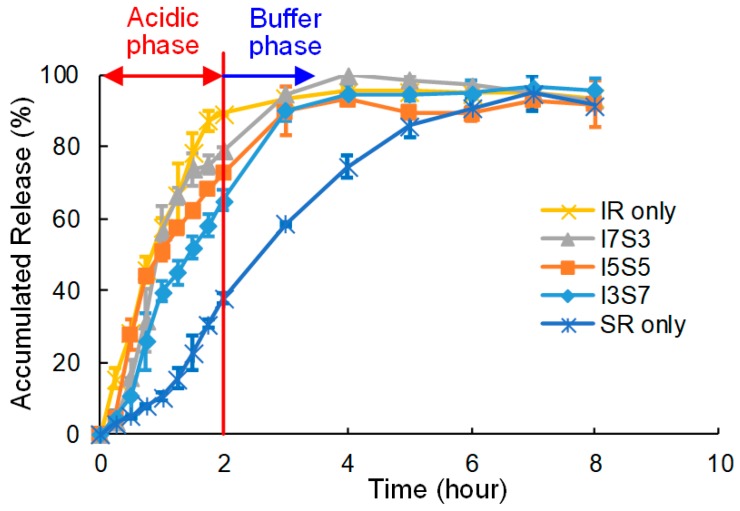
The result of the dissolution test.

The results of the dissolution test are shown in Figure 8. In the first two hours, during the acidic phase, the plot shows a faster dissolution rate when the mixture contains higher IR. “IR only” shows more than 85% dissolution in the first two hours, while “SR only” shows less than 40% in the same period. I3S7, I5S5 and I7S3 also show a similar pattern, that faster dissolution is caused by the IR composition. From the buffer phase, SR shows a linear dissolution during 2–3 h. By 4 h, all capsules except SR only reached nearly 100% dissolution, and beyond that the changes in measured dissolution are within the measurement error.

## 4. Discussion

**Figure 9:**
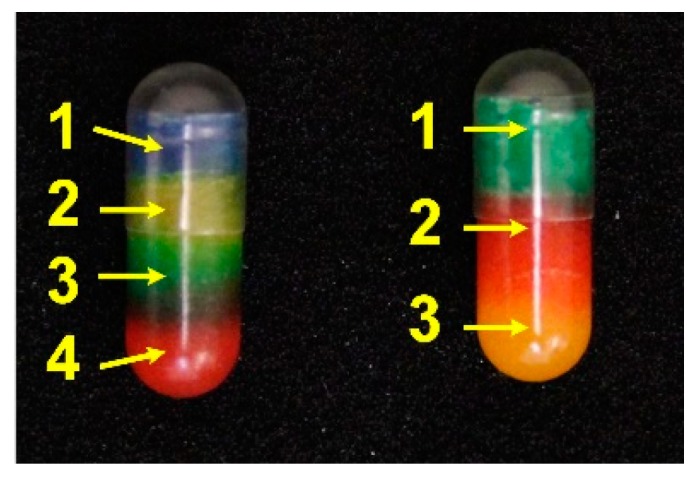
Examples of different layered capsules. Each color represents the drug. (Left) total of four layers; (right) total of three layers.

The goal of this study was to improve the throughput of fabricating capsules with various combinations of drugs for personalized medicine. This study demonstrated the feasibility of using the proposed coaxial extrusion setup to fabricate personalized capsules with a significantly faster production rate. Using this extrusion-based fabrication method, the extrusion process for one capsule takes 15 seconds. Considering loading/unloading and capping the capsule, the total fabrication time for one capsule is estimated around 20 seconds; the production rate is estimated as three capsules per minute, or 180 capsules per hour. This production rate is around three to eighteen times faster than the current 3D printing methods [13]. With the assumption that a patient is prescribed to take three capsules per day with a two weeks dose period, the total manufacturing time for this refill is around 14 minutes. There is some technique that also provides a fast production rate [33], however the fabrication setup proposed in this study provides multiple material choices with a cheaper equipment cost. This can bring the fabrication time of personalized drugs to a clinically relevant speed. While the current setup is limited to two materials, the setup can be modified to include more than two materials. Figure 9 shows capsules with four and three combinations of materials. 

Regarding to the equipment, the proposed fabrication setup only requires one axis of movement, as opposed to three axes required by 3D printers. The use of the coaxial needle setup has advantages over the use of a two-needle system on the easy alignment of needle in the capsule, a simpler system without the need of a second motion stage to switch between two needles, and faster processing. The multi-axis moving system is usually the most expensive component in a 3D printer. Reducing the moving component to a single axis not only lowers the price of the equipment, but also improves the reliability of the system. 

As mentioned in the introduction, one of the major challenges to personalized medication is the price [14], especially in the fabrication of a personal drug expected to be done at the point of care. We believe the cheaper initial price of the equipment and the faster fabrication speed would provide a more feasible option for fabricating personalized drugs.

In the present study, our group assessed the new fabrication system design for personalized medicine. The material we have chosen for this study showed a clear difference in porosity at SEM analysis, as Figure 7 indicates. Though the drug release profile is affected by many other factors, such as the size and distribution of particles, Figure 8 indicates it is possible to control the drug release profile by controlling the amount of material with our design; SR only and IR only showed the clear difference and its potential. As the acidic phase indicates, we were able to control the amount of drug released in a certain time frame by controlling the ratio of different materials, SR and IR. However, as ASA also hydrolyzes to salicylic acid (SA) in water [42], after two hours, at the buffer phase, the graph does not clearly show cumulative dissolution over time. It is possible that the capsules were still releasing of ASA, but at the same time, released ASA was hydrolyzing to SA, so that the measured accumulated release did not show a significant increase after the 3-hour mark for most cases. 

For this study, the material was chosen by proving the feasibility of the manufacturing method with the popular active drug, Aspirin. As the extrusion test and viscosity test indicates, the mixture was extruded successfully at the temperature of 50 °C to 60 °C. There is no need to change the parameter between SR-A, -B, -C or IR-A, -B, -C, because they are predetermined by the previous step. In summary, manufacturing techniques and goals have been achieved, but better material selection is required for increasing control of the drug release profile. 

There are three questions remaining for future work. First, it is unclear how a complex geometry effects on drug release kinetics. This study did not consider how the shape and geometry of each material both impact on drug release kinetics. A more complex structure, such as alternating layers of two materials, may provide different drug release kinetics, even though the overall composition is the same. The effect of internal structure can be further studied. Second, on the manufacturing side, how to increase the manufacturing capability of more complex structures, using the same setup, needs to be investigated. For example, a previous study indicates that it is possible to fabricate multi-layer tubular structures with controlled different layer thickness using a coaxial needle [43]. In this case, the outer needle extrudes the shell material, which effectively works as the capsule, and the inner needle extrudes the core material(s). It is expected that by controlling the wall thickness of the core-shell structure as a capsule would provide enough flexibility to control drug release kinetics. Additional material characterization tests, such as differential scanning calorimeter (DSC) and X-ray powder diffraction (XRPD), can be performed to examine how the drugs are incorporated into the excipients using the proposed process. The use of a capsule can also be potentially eliminated with using material which solidifies instantly with a mold. Third, extending the proposed setup and process to other materials or mixtures is still needed. This work includes an evaluation of how excipients incorporated with other characterization methods than just the drug release test. This coaxial extrusion process is very similar to the hot melt extrusion process which is commonly used for pharmaceutical manufacturing [44]. However, the hot melt extrusion process does not offer the flexibility of fabricating personalized drugs. Therefore, it is very possible to expand the proposed process to other drugs or excipient materials. 

## 5. Conclusions

In this study, we have demonstrated a new process to increase the feasibility of personalized medication, as well as designed new equipment for this process that is both simpler and more cost effective than current 3D printing methods. Thus, we believe that this study shows a clear potential to step toward the feasibility of personalized medication for future. The next step of this study would be the development of complex geometry, such as the core and shell, or embodied multiple cores, which will provide more diverse options for a complex drug release profile. 

## Figures and Tables

**Table 1:** The composition (%, *w*/*w*) of each material for the immediate release (IR) mixture.

IR Component	IR-A	IR-B	IR-C
HPMC2910	5	10	15
PEG	85	80	75
ASA	10	10	10

**Table 2:** The composition (%, *w*/*w*) of each material for the sustained release (SR) mixture.

SR Component	SR-A	SR-B	SR-C
HPMC2208	10	15	20
PEG	75	70	65
PAA	5	5	5
ASA	10	10	10

**Table 3:** The volume ratio of drug release testing (Unit: CC).

Ratio (volume/volume)	IR-C	SR-C
SR only	0.0	0.9
I3S7 (3:7)	0.27	0.63
I5S5 (5:5)	0.45	0.45
I7S3 (7:3)	0.63	0.27
IR only	0.9	0.0

## References

[1] Hamburg M.A. (2010). The Path to Personalized Medicine. N. Engl. J. Med..

[2] Holmes M.V., Shah T., Vickery C., Smeeth L., Hingorani A.D., Casas J.P. (2009). Fulfilling the promise of personalized medicine? Systematic review and field synopsis of pharmacogenetic studies. PLoS ONE.

[3] Kalia M. (2015). Biomarkers for personalized oncology: Recent advances and future challenges. Metabolism.

[4] Ginsburg G. (2001). Personalized medicine: Revolutionizing drug discovery and patient care. Trends Biotechnol..

[5] Kalia M. (2013). Personalized oncology: Recent advances and future challenges. Metabolism.

[6] Ma Q., Lu A.Y.H. (2011). Pharmacogenetics, pharmacogenomics, and individualized medicine. Pharmacol. Rev..

[7] Lesko L.J. (2007). Personalized medicine: Elusive dream or imminent reality?. Clin. Pharmacol. Ther..

[8] Mathur S., Mathur S., Sutton J., Sutton J. (2017). Personalized medicine could transform healthcare (Review). Biomed. Rep..

[9] Kantae V., Krekels E.H.J., Esdonk M.J.V., Lindenburg P., Harms A.C., Knibbe C.A.J., Van der Graaf P.H., Hankemeier T. (2017). Integration of pharmacometabolomics with pharmacokinetics and pharmacodynamics: Towards personalized drug therapy. Metabolomics.

[10] Cohen J.S. (1999). Ways to minimize adverse drug reactions: Individualized doses and common sense are key. Postgrad. Med..

[11] Tamargo J., le Heuzey J.-Y., Mabo P. (2015). Narrow therapeutic index drugs: A clinical pharmacological consideration to flecainide. Eur. J. Clin. Pharm..

[12] Yu L.X., Jiang W., Zhang X., Lionberger R., Makhlouf F., Schuirmann D.J., Muldowney L., Chen M.L., Davit B., Conner D. (2015). Novel bioequivalence approach for narrow therapeutic index drugs. Clin. Pharmacol. Ther..

[13] Burns M. (1999). Management of Narrow Therapeutic Index Drugs. J. Thromb. Thrombolysis.

[14] Lim S.H., Kathuria H., Tan J.J.Y., Kang L. (2018). 3D printed drug delivery and testing systems—A passing fad or the future?. Adv. Drug Deliv. Rev..

[15] Liang K., Brambilla D., Leroux J.-C. (2018). Is 3d printing of pharmaceuticals a disruptor or enabler?. Adv. Mater..

[16] Boukouvala F., Niotis V., Ramachandran R., Muzzio F.J., Ierapetritou M.G. (2012). An integrated approach for dynamic flowsheet modeling and sensitivity analysis of a continuous tablet manufacturing process. Comput. Chem. Eng..

[17] Skowyra J., Pietrzak K., Alhnan M.A. (2015). Fabrication of extended-release patient-tailored prednisolone tablets via fused deposition modelling (FDM) 3D printing. Eur. J. Pharm. Sci..

[18] Visser J.C., Woerdenbag H.J., Hanff L.M., Frijlink H.W. (2017). Personalized medicine in pediatrics: The clinical potential of orodispersible films. AAPS PharmSciTech.

[19] Wening K., Breitkreutz J. (2011). Oral drug delivery in personalized medicine: Unmet needs and novel approaches. Int. J. Pharm..

[20] Sandler N., Määttänen A., Ihalainen P., Kronberg L., Meierjohann A., Viitala T., Peltonen J. (2011). Inkjet printing of drug substances and use of porous substrates-towards individualized dosing. J. Pharm. Sci..

[21] Ozeki Y., Ando M., Watanabe Y., Danjo K. (2004). Evaluation of novel one-step dry-coated tablets as a platform for delayed-release tablets. J. Control. Release.

[22] Hosseini A., Körber M., Bodmeier R. (2013). Direct compression of cushion-layered ethyl cellulose-coated extended release pellets into rapidly disintegrating tablets without changes in the release profile. Int. J. Pharm..

[23] Tahara K., Yamamoto K., Nishihata T. (1995). Overall mechanism behind matrix sustained release (SR) tablets prepared with hydroxypropyl methylcellulose 2910. J. Control. Release.

[24] Norman J., Madurawe R.D., Moore C.M.V., Khan M.A., Khairuzzaman A. (2017). A new chapter in pharmaceutical manufacturing: 3D-printed drug products. Adv. Drug Deliv. Rev..

[25] Goyanes A., Buanz A.B.M., Basit A.W., Gaisford S. (2014). Fused-filament 3D printing (3DP) for fabrication of tablets. Int. J. Pharm..

[26] Goyanes A., Fina F., Martorana A., Sedough D., Gaisford S., Basit A.W. (2017). Development of modified release 3D printed tablets (printlets) with pharmaceutical excipients using additive manufacturing. Int. J. Pharm..

[27] Goyanes A., Wang J., Buanz A., Martínez-Pacheco R., Telford R., Gaisford S., Basit A.W. (2015). 3d printing of medicines: Engineering novel oral devices with unique design and drug release characteristics. Mol. Pharm..

[28] Goyanes A., Buanz A.B.M., Hatton G.B., Gaisford S., Basit A.W. (2015). 3D printing of modified-release aminosalicylate (4-ASA and 5-ASA) tablets. Eur. J. Pharm. Biopharm..

[29] Goyanes A., Allahham N., Trenfield S.J., Stoyanov E., Gaisford S., Basit A.W. (2019). Direct powder extrusion 3D printing: Fabrication of drug products using a novel single-step process. Int. J. Pharm..

[30] Genina N., Fors D., Vakili H., Ihalainen P., Pohjala L., Ehlers H., Kassamakoc I., Haeggström E., Vuorela P., Peltonen J. (2012). Tailoring controlled-release oral dosage forms by combining inkjet and flexographic printing techniques. Eur. J. Pharm. Sci..

[31] Khaled S.A., Burley J.C., Alexander M.R., Roberts C.J. (2014). Desktop 3D printing of controlled release pharmaceutical bilayer tablets. Int. J. Pharm..

[32] Rowe C.W., Katstra W.E., Palazzolo R.D., Giritlioglu B., Teung P., Cima M.J. (2000). Multimechanism oral dosage forms fabricated by three dimensional printingE. J. Control. Release.

[33] Goyanes A., Madla C.M., Umerji A., Piñeiro G.D., Montero J.M.G., Diaz M.J.L., Barcia M.G., Taherali F., Sánchez-Pintos P., Couce M.L. (2019). Automated therapy preparation of isoleucine formulations using 3D printing for the treatment of MSUD: First single-centre, prospective, crossover study in patients. Int. J. Pharm..

[34] Ishikawa T., Watanabe Y., Takayama K., Endo H., Matsumoto M. (2000). Effect of hydroxypropylmethylcellulose (HPMC) on the release profiles and bioavailability of a poorly water-soluble drug from tablets prepared using macrogol and HPMC. Int. J. Pharm..

[35] Lin H.-R., Chen Y.-T., Wu Y.-C., Lin Y.-J. (2018). Glycol chitin/PAA hydrogel composite incorporated bio-functionalized PLGA microspheres intended for sustained release of anticancer drug through intratumoral injection. J. Biomater. Sci. Polym. Ed..

[36] Pawar H., Douroumis D., Boateng J.S. (2012). Preparation and optimization of PMAA–chitosan–PEG nanoparticles for oral drug delivery. Colloids Surf. B Biointerfaces.

[37] Ban C., Jo M., Lim S., Choi Y.J. (2018). Control of the gastrointestinal digestion of solid lipid nanoparticles using PEGylated emulsifiers. Food Chem..

[38] Wang J., Goyanes A., Gaisford S., Basit A.W. (2016). Stereolithographic (SLA) 3D printing of oral modified-release dosage forms. Int. J. Pharm..

[39] Abd-Allah F.I., Dawaba H.M., Ahmed A.M.S. (2010). Preparation, characterization, and stability studies of piroxicam- loaded microemulsions in topical formulations. Drug Discov..

[40] Alexander A., Ajazuddin, Khan J., Saraf S., Saraf S. (2014). Polyethylene glycol (peg)–poly(n-isopropylacrylamide) (pnipaam) based thermosensitive injectable hydrogels for biomedical applications. Eur. J. Pharm. Biopharm..

[41] Torrado S., Cadorniga R., Torrado J.J. (1996). Effect of drug release rate on bioavailability of different aspirin tablets. Int. J. Pharm..

[42] Barry E., Borer L.L. (2000). Experiments with aspirin. J. Chem. Educ..

[43] Yu I., Chen R., Grindrod S. Fabrication of gellan gum tubular structure using coaxial needles: A study on wall thickness and encapsulation. Proceedings of the ASME 2018 13th International Manufacturing Science and Engineering Conference.

[44] Crowley M.M., Zhang F., Repka M.A., Thumma S., Upadhye S.B., Battu S.K., McGinity J.W., Martin C. (2007). Pharmaceutical Applications of Hot-Melt Extrusion: Part I. Drug Dev. Ind. Pharm..

